# Glucose-Assisted Synthesis of In_2_O_3_ Nanorods for High-Performance Ozone Detection

**DOI:** 10.3390/nano16060366

**Published:** 2026-03-17

**Authors:** Xiumei Xu, Yi Zhou, Haijiao Zhang, Mengmeng Dai, Gui Wang, Gang Yang, Yongsheng Zhu

**Affiliations:** 1 College of Materials and New Energy, Nanyang Normal University, 1638 Wolong Road, Nanyang 473061, China; 20142007@nynu.edu.cn; 2 College of Physics and Electronic Engineering, Nanyang Normal University, 1638 Wolong Road, Nanyang 473061, China; 2024085401005@nynu.edu.cn (Y.Z.); 20252034@nynu.edu.cn (M.D.); guiwang@nynu.edu.cn (G.W.); yanggang@nynu.edu.cn (G.Y.)

**Keywords:** In_2_O_3_ nanorods, glucose-assisted synthesis, ozone gas sensors, morphology control

## Abstract

In_2_O_3_ has high electron mobility, strong affinity for oxidizing gases, and abundant tunable surface oxygen species. These features enable efficient charge transfer during ozone adsorption, making In_2_O_3_ a promising ozone-sensing material. However, conventional In_2_O_3_-based gas sensors still suffer from insufficient sensitivity at low ozone concentrations and slow response/recovery rates, limiting their performance for high-precision gas detection. In this study, morphology-controlled In_2_O_3_ nanorods were synthesized via a glucose-assisted hydrothermal method, enabling coordinated regulation of the material structure and surface properties. Compared with conventional In_2_O_3_ nanocubes, the glucose-modulated In_2_O_3_ nanorods exhibited an approximately sevenfold increase in response toward 1 ppm O_3_, indicating markedly improved capability for detecting low-concentration ozone. In addition, the sensor demonstrated a relatively low detection limit of about 80 ppb and fast response/recovery behavior (108 s/238 s). This strategy improves gas sensing performance through morphology optimization, increased surface active sites, and enhanced electron transport, offering a feasible materials design route for high-performance ozone gas sensors and showing potential for real-time environmental ozone monitoring and related applications.

## 1. Introduction

Ozone, a highly reactive oxidative substance, is widely used in medical device sterilization, hospital disinfection, and food preservation, while also representing an important environmental pollutant whose real-time monitoring is crucial for air quality management and public health [1,2,3,4]. Metal oxide semiconductor (MOS) gas sensors are considered promising candidates for ozone detection because of their portability, low cost, and rapid response [5]. In particular, In_2_O_3_-based sensors have attracted attention due to their high electron mobility and strong affinity toward oxidizing gases [6,7]. Nevertheless, conventional In_2_O_3_ sensors often suffer from low sensitivity at trace ozone concentrations and slow response/recovery rates [8,9]. They typically require elevated operating temperatures (150–350 °C), which not only increases power consumption and fabrication complexity but also makes the sensors susceptible to humidity interference, limiting their practical use in low-concentration, real-time monitoring applications [10,11].

To overcome these limitations, various strategies have been explored, including noble metal loading, defect engineering, and surface functionalization. For instance, Au or Pd loading can enhance electron transport and gas adsorption, improving sensor response while lowering the operating temperature [12]. Introducing oxygen vacancies or surface defects can generate additional active sites, promoting gas adsorption and charge transfer [13]. Despite these advancements, developing a low-cost, facile, and efficient strategy that simultaneously improves sensitivity, lowers detection limits, and accelerates response/recovery at moderate temperatures remains a key challenge for high-performance ozone sensing [14].

To address the limitations of conventional In_2_O_3_ in low-concentration ozone detection, a glucose-assisted hydrothermal route was adopted in this work to tailor the morphology of In_2_O_3_ into nanorods. By comparing the resulting nanorods with traditional In_2_O_3_ nanocubes, the influence of morphology on ozone-sensing behavior was systematically evaluated. The results demonstrate that morphology engineering via glucose assistance is an effective strategy to improve the overall sensing performance of In_2_O_3_-based ozone sensors.

## 2. Experimental Section

### 2.1. Chemical Materials

Indium nitrate hydrate (In(NO_3_)_3_·xH_2_O, 99.9%), glucose (99.5%) and urea (99.5%) were obtained from Shanghai Aladdin Biochemical Technology Co., Ltd., Shanghai, China. In addition, absolute ethanol and deionized (DI) water were used for all of the experiments. All reagents were analytical grade and used as purchased without further purification.

### 2.2. Synthesis of In_2_O_3_ Nanorods (S1)

**Figure 1:**
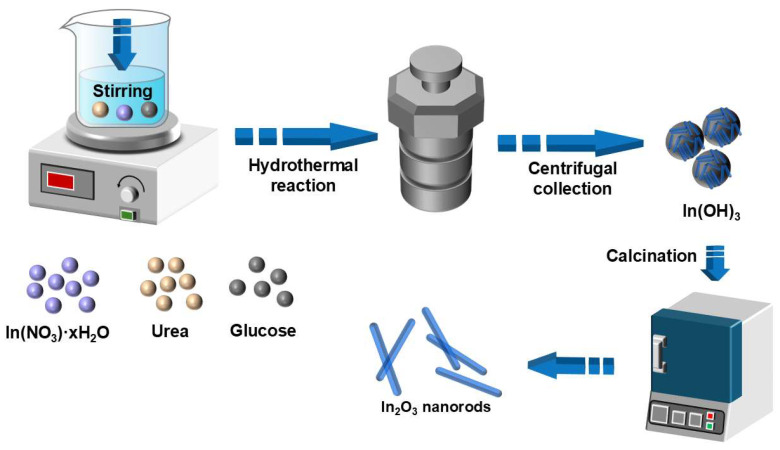
Schematic of synthesis of In_2_O_3_ nanorods.

Figure 1 illustrates the schematic procedure for the preparation of In_2_O_3_ nanorods (S1). Glucose was employed as a morphology-directing agent to assist in the formation of a hydroxide-based indium precursor via a straightforward hydrothermal method, which was subsequently calcined to obtain S1. In a typical synthesis, 0.6 g of In(NO_3_)_3_·xH_2_O was dispersed in 72 mL of deionized water and stirred at 60 °C until completely dissolved. Then, 0.3 g of urea and 0.9 g of glucose were added, and the mixture was stirred for 1 h. The solution was transferred to a 100 mL Teflon-lined autoclave and heated at 160 °C for 12 h. After naturally cooling to room temperature, the precipitate was collected by centrifugation and thoroughly washed several times with deionized water and absolute ethanol until the supernatant became clear. The product was dried in an oven at 60 °C for 24 h and then calcined in a muffle furnace at 550 °C for 2 h to obtain In_2_O_3_ nanorods (S1). For comparison, pristine In_2_O_3_ nanocubes (S2) were synthesized under the same experimental conditions, except that glucose was omitted from the reaction system.

### 2.3. Material Characterization

XRD patterns were analyzed by X-ray diffractometer (Rigaku Corporation, Tokyo, Japan) equipped with Cu Kα radiation (λ = 1.5406 Å). X-ray photoelectron spectroscopy (XPS) measurements were performed by a Thermo Scientific K-Alpha (Thermo Fisher Scientific, Waltham, MA, USA). The specific surface area and pore distribution of the samples were measured by Micromeritics ASAP 2460 (Micromeritics Instrument Corporation, Norcross, GA, USA). The nanoscale morphology of the sample was observed using a field-emission scanning electron microscope (SEM) model JSM-7800F (JEOL Ltd., Tokyo, Japan) and a transmission electron microscope (TEM) model JEM-F200 (JEOL Ltd., Tokyo, Japan).

## 3. Results and Discussion

### 3.1. Structure and Morphology Characterization

**Figure 2:**
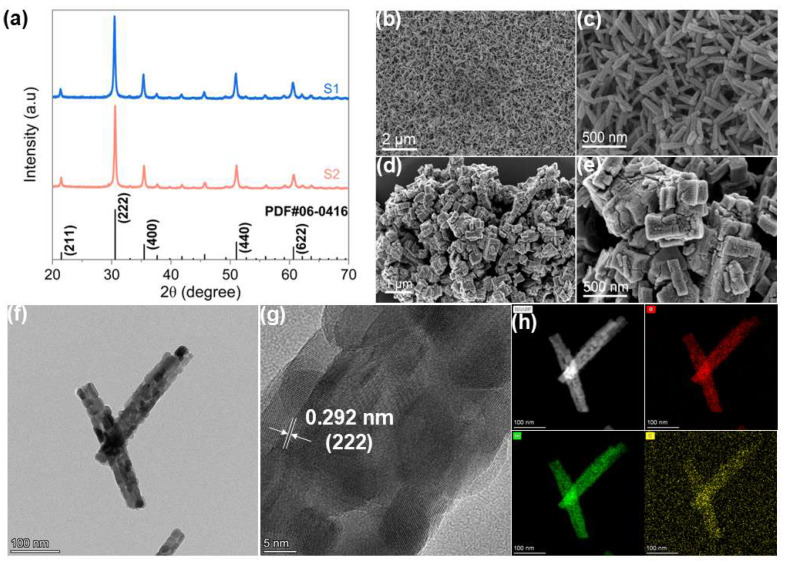
(**a**) XRD patterns of the samples. SEM images of S1 (**b**,**c**) and S2 (**d**,**e**), (**f**) TEM image of S1, (**g**) High-resolution TEM (HRTEM) image of S1, (**h**) Elemental mapping of S1 by EDS.

X-ray powder diffraction (XRD) was employed to confirm the crystal structure and phase composition of the prepared samples. As shown in Figure 2a, the XRD patterns of S1 and S2 exhibited distinct diffraction peaks at 21.4°, 30.6°, 35.5°, 51.0°, and 60.6°, which can be indexed to the (221), (222), (400), (440), and (622) planes of cubic In_2_O_3_ (JCPDS No. 06-0416), respectively. All observed peaks could be attributed to cubic In_2_O_3_, and no additional peaks corresponding to secondary phases were detected, indicating a high phase purity of the samples. The microstructure and morphology of the samples were systematically characterized by scanning electron microscopy (SEM) and transmission electron microscopy (TEM). As shown in Figure 2b–f and Figure S1a, in contrast to the nanocube aggregates observed in the sample synthesized without glucose, hierarchical nanorods in the S1 sample are composed of closely packed nanoparticles. The formation of this structure is likely related to the synergistic effect of glucose and urea during the hydrothermal process. Glucose can act as a structure-directing agent and stabilizer, regulating the nucleation and growth of indium species and providing favorable conditions for the formation of a hierarchical framework. Meanwhile, urea gradually decomposes during the hydrothermal reaction to release OH^−^ ions, which react with In^3+^ to form In(OH)_3_ nuclei. As the number of nuclei increases, these primary nanoparticles tend to undergo self-assembly and aggregation to reduce surface energy, gradually forming nanorod structures composed of closely packed nanoparticles. Subsequent thermal treatment converts the precursor into crystalline In_2_O_3_ while largely preserving the hierarchical nanorod morphology [15]. High-resolution TEM (HRTEM) images Figure 2g and Figure S1b reveal a lattice spacing of ~0.292 nm, corresponding to the (222) plane of cubic In_2_O_3_, indicating that both S1 and S2 maintain well-preserved crystalline structures. Figure 2h and Figure S1c–e present the EDS elemental mapping of S1 and S2, showing uniform distributions of In and O in S1. A small amount of C is also detected, and subsequent XPS analysis confirms that part of the carbon is incorporated into the In_2_O_3_ lattice, while the remainder is mainly located on the sample surface.

**Figure 3:**
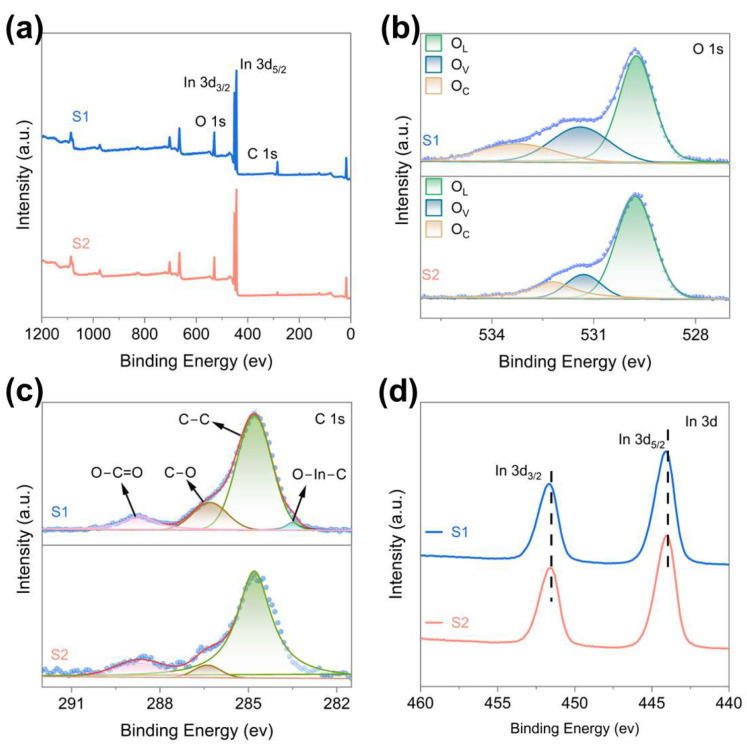
(**a**) XPS survey spectrum, (**b**) High-resolution O 1s spectrum, (**c**) High-resolution C 1s spectrum, (**d**) High-resolution In 3d spectrum.

To further elucidate the surface chemical states and elemental composition of the samples, X-ray photoelectron spectroscopy (XPS) measurements were performed. The survey spectra (Figure 3a) confirm that both S1 and S2 are primarily composed of In, O, and C elements. All binding energies were calibrated using the adventitious C 1s peak at 284.8 eV as a reference. High-resolution O 1s spectra (Figure 3b) were deconvoluted into three distinct components centered at 529.7, 531.4, and 533.3 eV, which can be assigned to lattice oxygen (O_L_), defect-related oxygen species associated with oxygen vacancies (O_V_), and surface-adsorbed oxygen (O_C_), respectively [16,17,18,19]. The quantitative distribution of these oxygen species is summarized in Table S1. Compared with sample S2 (O_V_ 14.3%; O_C_ 17.1%), sample S1 shows O_V_ and O_C_ proportions of 27% and 17.4%, respectively. Since oxygen vacancies are widely recognized as critical active sites for gas adsorption on metal-oxide semiconductors, their enrichment is expected to facilitate surface reactions with target gas molecules and potentially benefit gas sensing performance [20,21,22]. To clarify the chemical state of carbon, the C 1s spectra were also analyzed by peak fitting (Figure 3c). For both samples, three main components located at 284.8, 286.3, and 288.7 eV correspond to C–C, C–O, and C=O bonds, respectively [23,24,25]. Notably, an additional weak feature at approximately 283.5 eV appears only in S1, which is commonly attributed to O-In-C bonding [23,26]. This suggests that carbon species may interact chemically with the In_2_O_3_ lattice rather than merely existing as surface contaminants. This result supports the inference that carbon is involved in lattice modulation. High-resolution In 3d spectra (Figure 3d) display two characteristic peaks at approximately 444.0 eV and 451.7 eV, corresponding to In 3d_5/2_ and In 3d_3/2_ of In 3d, respectively [27]. Compared with S2, both peaks in S1 shift perceptibly toward higher binding energies, indicating a change in the local electronic environment around indium atoms. This shift may arise from the combined effects of carbon–lattice interactions and the increased concentration of oxygen vacancies, which collectively alter local charge distribution [28,29,30].

**Figure 4:**
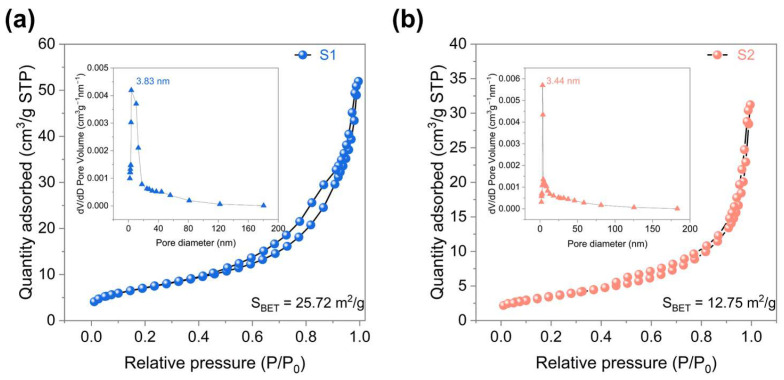
(**a**) N_2_ adsorption-desorption isotherm of S1 (inset: pore size distribution), and (**b**) N_2_ adsorption-desorption isotherm of S2 (inset: pore size distribution).

Nitrogen adsorption–desorption isotherms of both samples (Figure 4a,b) exhibit clear hysteresis loops over a wide relative pressure range, confirming their mesoporous nature. S2 shows a pore size distribution centered at ~3.44 nm with a specific surface area of 12.75 m^2^/g, whereas S1 presents slightly larger pores (~3.83 nm) and a substantially higher surface area of 25.72 m^2^/g. Such structural features are favorable for gas diffusion and adsorption, likely exposing more active sites and increasing the proportion of surface oxygen species. Consequently, the synergistic combination of abundant oxygen vacancies and enhanced surface area suggests that S1 has strong potential for improved gas sensing performance [31].

### 3.2. Sensing Properties

**Figure 5:**
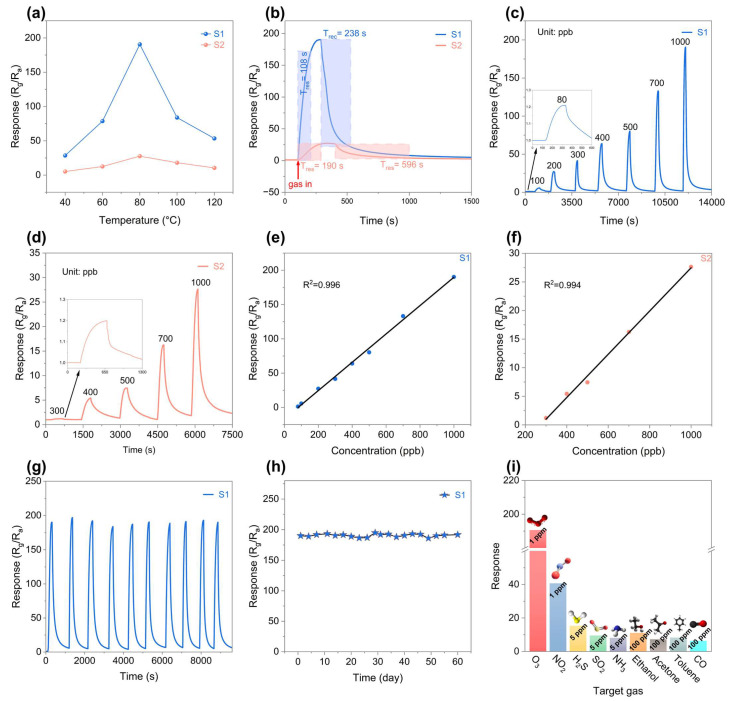
Gas sensing performance of the sensors measured at an operating temperature of 80 °C unless otherwise stated. (**a**) Response of all sensors to 1 ppm O_3_ at different operating temperatures. (**b**) Response/recovery curves of all sensors toward 1 ppm O_3_. (**c**,**d**) Dynamic response curves of the S1 and S2 sensors to various O_3_ concentrations. (**e**,**f**) Linear fitting between sensor responses and O_3_ concentrations for the S1 and S2 sensors. (**g**) Repeatability of the S1 sensor toward 1 ppm O_3_. (**h**) Long-term stability of the S1 sensor toward 1 ppm O_3_. (**i**) Responses of the S1 sensor to various kinds of detected gases.

The synthesized samples were ground into a uniform slurry and coated onto the outer surface of commercial ceramic tubes, which were subsequently mounted onto sensor bases for gas-sensing measurements. Detailed procedures can be found in Supporting Information and the testing procedure and schematic illustration of the sensor configuration are shown in Figure S2 [32]. To determine the optimal working temperature, the responses of both sensors to 1 ppm O_3_ were measured across 40–120 °C (Figure 5a). Both S1 and S2 exhibited a volcano-type temperature dependence, reaching a maximum response at 80 °C, which was selected as the optimal operating temperature for subsequent tests. At 80 °C, S1 showed a response of 190.6 to 1 ppm O_3_, significantly higher than S2 (27.6). The dynamic response/recovery curves at 80 °C are shown in Figure 5b. The response time (T_res_) and recovery time (T_rec_) of S1 toward 1 ppm O_3_ were 108 s and 238 s, respectively, significantly shorter than those of S2 (190 s and 596 s). The concentration-dependent responses of S1 and S2 to O_3_ are presented in Figure 5c,d. The response of both sensors increased monotonically with O_3_ concentration, and the sensors quickly returned to baseline upon gas removal. Notably, S1 exhibited a measurable response even at 80 ppb, indicating potential for low-concentration ozone detection. Linear regression analysis (Figure 5e) gave a correlation coefficient of 0.996 for S1, confirming excellent linearity. Based on a practical response threshold of S ≥ 1.2 [33], the detection limit of S1 was estimated to be ~80 ppb. In contrast, S2 showed a correlation coefficient of 0.994 and a higher detection limit of ~300 ppb (Figure 5f). The enhanced gas sensing performance of S1 can be attributed to the hierarchical nanorod structure formed through the glucose-assisted hydrothermal strategy. These nanorods are composed of tightly packed nanoparticles rather than dense single-crystal structures, resulting in abundant interparticle interfaces. Such a structure not only significantly increases the specific surface area and provides a large number of active sites for gas adsorption and reaction, but also constructs plentiful internal mesoporous channels, which facilitate rapid gas diffusion and promote the surface reaction kinetics between O_3_ molecules and adsorbed oxygen species. Therefore, compared with S2, the sensor exhibits a higher response value as well as faster response/recovery characteristics [34,35]. Sensor repeatability and long-term stability were assessed by conducting ten consecutive air-O_3_-air cycles at 80 °C. As shown in Figure 5g, S1 exhibited nearly identical response/recovery profiles over all cycles, confirming high reproducibility. The response variation in S1 to 1 ppm O_3_ over 60 days is presented in Figure 5h, indicating negligible drift and excellent long-term stability, suitable for continuous monitoring applications. Selectivity tests were performed at 80 °C using 1 ppm O_3_, 1 ppm NO_2_, 5 ppm SO_2_, H_2_S, NH_3_, and 100 ppm ethanol, acetone, toluene, and CO (Figure 5i). S1 demonstrated a substantially higher response to O_3_ (190.6) than to all other interfering gases, confirming its superior ozone selectivity. The enhanced sensing performance of S1 toward O_3_ can be attributed to both the intrinsic chemical properties of ozone and the structural characteristics of the sensing material. Ozone is a triatomic molecule with a high oxidation potential and large electron affinity; therefore, it can more readily adsorb and dissociate on the sensor surface compared with interfering gases such as NO_2_. This process extracts a large number of electrons from the conduction band of the sensing material, resulting in a pronounced change in resistance [36]. Meanwhile, the hierarchical nanorod structure composed of closely packed nanoparticles facilitates gas diffusion and provides abundant active reaction sites, further amplifying the selective response toward O_3_. As a result, the nanorod-structured In_2_O_3_ exhibits superior ozone sensing performance [37]. It should be noted that some gases exhibit incomplete recovery at relatively low operating temperatures. This phenomenon can be attributed to the slow desorption kinetics of gas molecules on the sensing surface. At low temperatures, the desorption rate of adsorbed species decreases significantly, resulting in prolonged recovery or even partial irreversible adsorption behavior (Figure S3) [38]. Moreover, compared with materials reported in other studies, the performance of the S1 sensor is also superior (Table S2).

### 3.3. Sensing Mechanism

**Figure 6:**
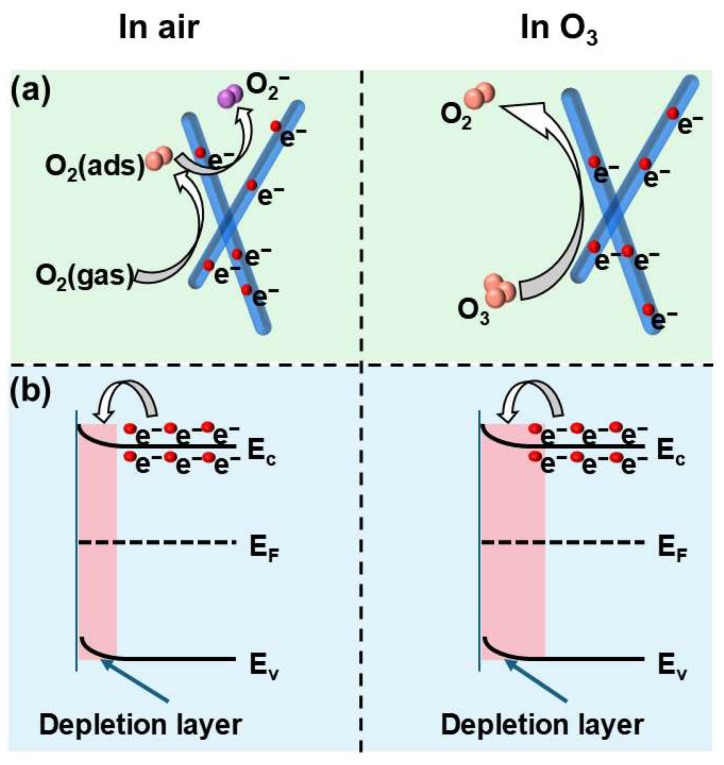
Schematic illustration of the gas-sensing mechanism of the S1 sensor under air and O_3_ atmospheres: (**a**) Surface adsorption and charge-transfer processes; (**b**) Corresponding energy band structures and depletion layers.

The gas sensing behavior of the In_2_O_3_ nanorods synthesized via the glucose-assisted hydrothermal method follows the typical mechanism of metal oxide semiconductor sensors, which is governed by surface charge transfer processes between adsorbed gas molecules and the sensing material (Figure 6a) [39]. When exposed to air, oxygen molecules are first adsorbed on the surface of In_2_O_3_ and capture electrons from the conduction band, forming ionized oxygen species (Equations (1) and (2)) [40]. This electron extraction leads to the formation of an electron depletion layer near the surface, resulting in an increase in the sensor resistance.

(1) $$O_{2(gas)}\to O_{2(ads)}$$

(2) $$O_{2(ads)}+e^{-}\to O_{2(ads)}^{-}$$

The band structure of In_2_O_3_ is illustrated in Figure 6b. Upon exposure to ozone, the highly oxidizing O_3_ molecules interact with the adsorbed oxygen species and directly capture electrons from the conduction band of In_2_O_3_, leading to a further expansion of the surface depletion layer and a pronounced increase in resistance. The related reactions can be expressed as follows (Equations (3) and (4)) [41]:

(3) $$O_{3}+O_{2(ads)}^{-}+2e^{-}\to O_{2}+3O^{-}$$

(4) $$O_{3}+e^{-}\to O_{2}+O^{-}$$

It is worth noting that the formation of this hierarchical nanorod structure is closely related to the glucose-assisted hydrothermal process. During the synthesis, glucose acts as a structure-directing and stabilizing agent, regulating the nucleation and growth of indium species and promoting the self-assembly of primary nanoparticles into nanorod architectures. Furthermore, the superior sensing performance of S1 can also be attributed to its hierarchical nanorod structure composed of closely packed nanoparticles. This particle-assembled architecture introduces abundant interparticle interfaces and mesoporous channels, which facilitate rapid gas diffusion and improve the accessibility of active sites. Meanwhile, the larger specific surface area provides more reaction sites for the adsorption of oxygen and ozone molecules. XPS analysis further reveals that S1 possesses a higher concentration of oxygen vacancies than S2 (Figure 3b). These oxygen vacancies act as important active sites and promote charge transfer during the sensing reaction [8]. Therefore, the synergistic effects of the hierarchical nanorod structure, abundant oxygen vacancies, and large specific surface area accelerate the surface reaction kinetics and induce a more pronounced resistance change upon exposure to ozone [42,43,44].

## 4. Conclusions

In this study, In_2_O_3_ nanorods (S1) were successfully synthesized via a glucose-assisted hydrothermal method. Structural characterizations revealed that S1 closely packed nanoparticles assembled into hierarchical nanorods, generating abundant interparticle interfaces. Gas-sensing tests showed that S1 exhibited a high response of 190.6 to 1 ppm O_3_, a low detection limit of approximately 80 ppb, and fast response/recovery times (T_res_ = 108 s and T_rec_ = 238 s), significantly outperforming pristine In_2_O_3_ nanocubes (S2). The enhanced performance is mainly attributed to microstructural features: the particle-assembled architecture provides abundant interfaces, the increased surface area and mesoporous channels offer numerous active sites and facilitate gas diffusion, and the higher concentration of oxygen vacancies accelerates surface electron transfer. These synergistic effects collectively lead to higher response values and faster response/recovery kinetics compared with S2. Overall, the glucose-assisted morphology control strategy effectively tailors the microstructure of In_2_O_3_, providing a practical and scalable approach for designing high-performance metal-oxide semiconductor gas sensors, particularly suitable for environmental ozone monitoring.

## Data Availability

The original contributions presented in this study are included in the article/Supplementary Materials. Further inquiries can be directed to the corresponding authors.

## Supplementary Materials

The following supporting information can be downloaded at: https://www.mdpi.com/article/10.3390/nano16060366/s1, Figure S1: (a) TEM image of S2, (b) High-resolution TEM (HRTEM) image of S2, (c–e) Elemental mapping of S2 by EDS; Figure S2: Schematic of the sensors and the gas sensing test equipment; Figure S3: Response/recovery curves of In_2_O_3_ nanorods toward (a) NO_2_, (b) H_2_S, (c) SO_2_, (d) NH_3_, (e) Ethanol, (f) Acetone, (g) Toluene, and (h) CO at 80 °C; Table S1: Peak position and surface oxygen specie contents of the samples; Table S2: The comparison in O_3_ sensing performance of metal oxide-based sensors between the reported studies and our work [45,46,47].
