# Supplementary material for: Glucose-Assisted Synthesis of In2O3 Nanorods for High-Performance Ozone Detection
Source: Nanomaterials (Basel). 2026 Mar 17;16(6):366. doi: 10.3390/nano16060366 (PMC13029729; doi:10.3390/nano16060366)
Supplement: Supplementary file 1 [file nanomaterials-16-00366-s001.zip › nanomaterials-4179558-supplementary.pdf]

Supporting Information

Fabrication and Testing of the Sensor

The gas sensors were fabricated using a conventional coating technique. Specifically, 10 mg of the synthesized sample was dispersed in 2 mL of ethanol and thoroughly ground to obtain a uniform slurry. The resulting slurry was then coated onto the outer surface of a commercial ceramic tube equipped with a pair of Pt wires and two Au electrodes. The operating temperature of the sensor was controlled by a Ni-Cr alloy heater located inside the ceramic tube. After coating, the ceramic tube was placed in a muffle furnace and sintered at 200 °C for 2 h to ensure strong adhesion and effective electrical contact between the sensing layer and the ceramic substrate. Subsequently, the sintered ceramic tube and the Ni-Cr alloy heating wire were welded to the sensor base, and the entire device was aged in air at 400 °C for 48 h to further improve the long-term stability and repeatability of the sensor.

Gas sensing measurements were carried out in a static test system. The sensing chamber consisted of a sealed glass bottle with a volume of approximately 1 L. First, fresh air (atmospheric air) was introduced into a closed glass chamber, and then a given amount of the tested gas was injected into the chamber by a microinjector, and the sensor was put into the chamber for the measurement of the sensitive performance. The desired concentration of the VOC gas was obtained by the static liquid–gas distribution method, which was calculated by the following formula:

$$\mathrm{C} = \frac{22.4 \times \varphi \times \rho \times \mathrm{V}_1}{\mathrm{M} \times \mathrm{V}_2}$$

where C (ppm) is the target gas concentration, φ the required gas volume fraction, ρ (g/mL) the density of the liquid, V_1(L) the volume of liquid, V_2(L) the volume of the chamber, and M (g/mol) the molecular weight of the liquid. The ambient temperature during the tests was approximately 25 °C, and the relative humidity ranged from 30% to 40%. The sensor response was defined as the resistance ratio, expressed as Ra/Rg in

reducing gases and Rg/Ra in oxidizing gases, where Rg and Ra represent the resistance in the target gas and in air, respectively. The response and recovery times were defined as the time required for the sensor resistance to reach 90% of the total resistance change during the adsorption and desorption processes. Resistance signals were recorded using a UT8806 measurement system (UNI-TREND Technology Co., Ltd., Guangdong, China).

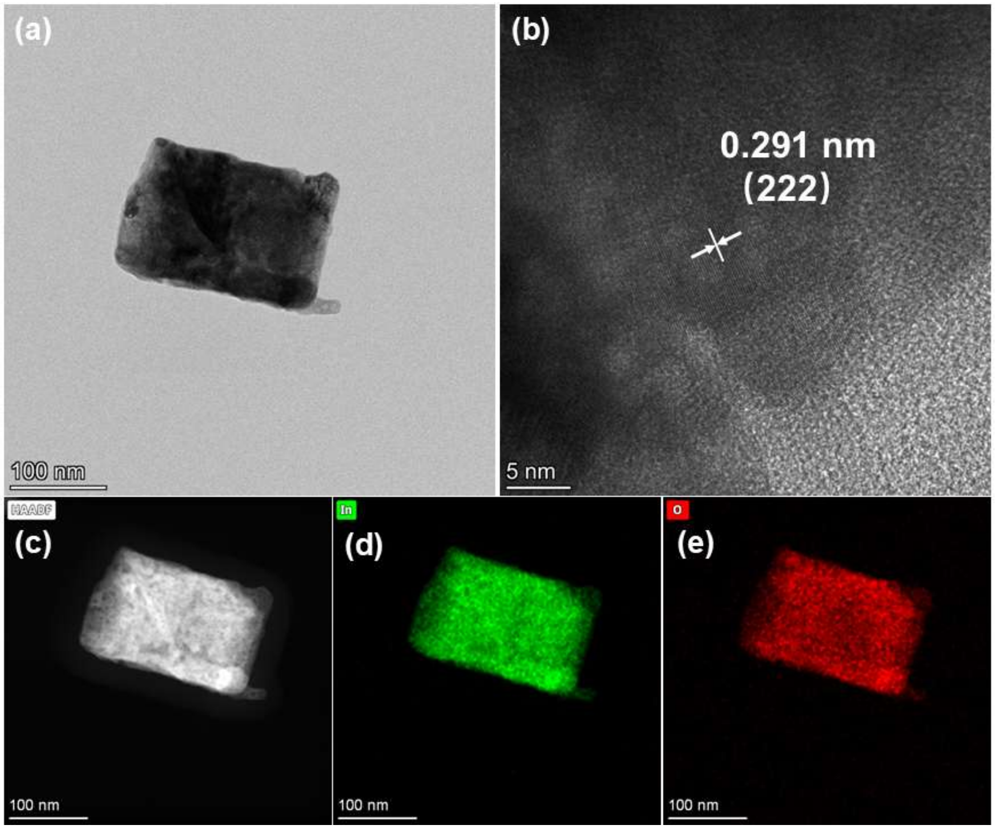

Figure S1 (**a**) TEM image of S2. (**b**) High-resolution TEM (HRTEM) image of S2. (**c-e)** Elemental mapping of S2 by EDS.

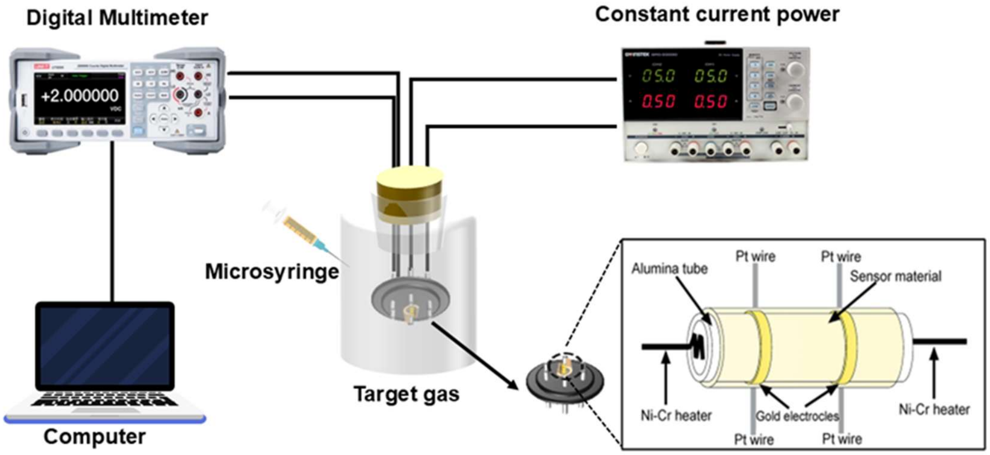

Figure S2. Schematic of the sensors and the gas sensing test equipment.

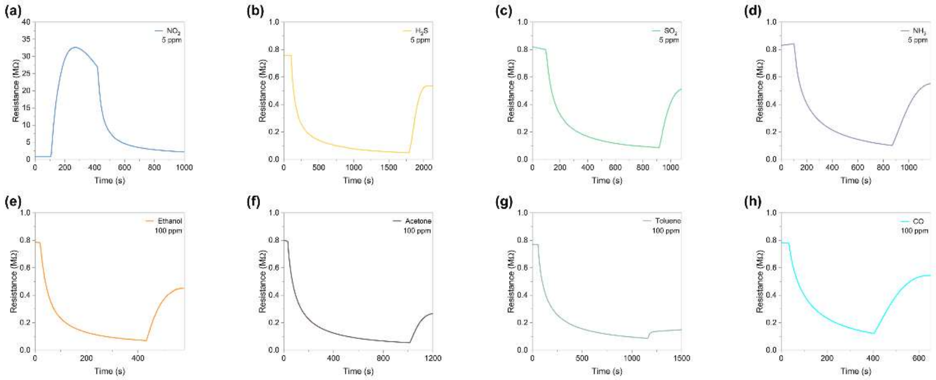

Figure S3. Response–recovery curves of In_2O_3 nanorods toward (**a**) NO_2, (**b**) H_2S, (**c**) SO_2, (**d**) NH_3, (**e**) Ethanol, (**f**) Acetone, (**g**) Toluene, and (**h**) CO at 80 °C.

Table S1. Peak position and surface oxygen species contents of the samples.

Samples	Lattice oxygen (O_L)	Oxygen vacancies (O_V)	Chemisorbed oxygen (O_C)
S1	529.7eV (55.6%)	531.4 eV (27 %)	533.3 eV (17.4%)
S2	529.8 eV (68.6%)	531.3 eV (14.3%)	532.2 eV (17.1%)

Table S2. The comparison in O_3 sensing performance of metal oxide-based sensors between the reported literature and our work.

Samples	Concentration	Response	Condition	LOD	Ref.
$CuAlO_2$	1.15 ppm	≈1.9	250 ºC	200 ppb	[45]
$ZnCo_2O_4$	0.89 ppm	71	200 ºC	80 ppb	[46]
SnO_2	1.3 ppm	1.2	n/a	400 ppb	[47]
S1	1 ppm	≈190.6	80 ºC	80 ppb	This work
